# CYP3A4*1B and CYP3A5*3 SNPs significantly impact the response of Egyptian candidates to high-intensity statin therapy to atorvastatin

**DOI:** 10.1186/s40001-024-02109-7

**Published:** 2024-11-10

**Authors:** Mohammed G. Maslub, Nur Aizati Athirah Daud, Mahasen A. Radwan, Abubakar Sha’aban, Arafa G. Ibrahim

**Affiliations:** 1 Clinical Pharmacy/Pharmacy Practice Department, Faculty of Pharmacy, Egyptian Russian University, Cairo-Suez Road, Badr City, 11829 Cairo Egypt; 2 School of Pharmaceutical Sciences, Universiti Sains Malaysia, 11800 Gelugor, Penang Malaysia; 3 Human Genome Centre, School of Medical Sciences, Universiti Sains Malaysia Health Campus, 16150 Kota Bharu, Kelantan Malaysia; 4 Division of Population Medicine, Cardiff University, Cardiff, Wales CF14 4YS UK; 5 Cardiology Department, Faculty of Medicine, Helwan University, Helwan City, 11795 Cairo Egypt

**Keywords:** Atorvastatin, CYP3A4*1B, CYP3A5*3, Genetic polymorphism, SNPs, Effectiveness, Safety, Egypt

## Abstract

**Background:**

A single nucleotide polymorphism (SNP) is a variation in the DNA sequence that results from the alteration of a single nucleotide in the genome. Atorvastatin is used to treat hypercholesterolemia. It belongs to a class of drugs called statins, which lower elevated levels of total cholesterol (TC) and low-density lipoprotein cholesterol (LDL-C). Research findings on the associations between the response to atorvastatin and genetic polymorphisms in *CYP3A4* and *CYP3A5* are inconclusive. The effects of CYP3A4*1B (*rs2740574* C/T) and CYP3A5*3 (*rs776746* T/C) on atorvastatin therapy have not been previously studied among Egyptians.

**Objective:**

This research aimed to investigate the effects of the genetic polymorphisms CYP3A4*1B and CYP3A5*3 on atorvastatin treatment in Egyptians.

**Methods:**

In this prospective cohort study, 100 subjects were genotyped for these SNPs. All participants were screened for serum lipid profiles, liver enzymes, total bilirubin (TB), and creatine kinase (CK) before and after 40 mg postatorvastatin therapy. Atorvastatin plasma levels were assessed posttreatment; atorvastatin pharmacokinetics were evaluated in five carriers of the CYP3A4*1B (T/T) and CYP3A5*3 (C/C) genotypes.

**Results:**

The allele frequencies of the CYP3A4*1B and CYP3A5*3 SNPs were 86% and 83%, respectively. The CYP3A4*1B (T/T) and CYP3A5*3 (C/C) genotypes significantly improved the serum triglyceride (TG) level (P < 0.05) and elevated the TB level (P < 0.001). Atorvastatin plasma levels were greater in CYP3A4*1B (T/T) (P < 0.05) and CYP3A5*3 (C/C) (P < 0.001) genotype carriers. Both SNPs significantly affected the pharmacokinetics of atorvastatin compared with those of Egyptian volunteers and various ethnic populations.

**Conclusions:**

The CYP3A4*1B and CYP3A5*3 variants were prevalent in the study participants and could impact the effectiveness and safety of atorvastatin therapy. The mutant genotype of the CYP3A4*1B SNP and the CYP3A5*3 SNP led to high atorvastatin levels. Both variants had a notable effect on the pharmacokinetics of atorvastatin among Egyptians compared with healthy Egyptians and volunteers from other ethnic populations. Overall, clinicians can learn more about the impact of both variants in response to atorvastatin.

**Graphical Abstract:**

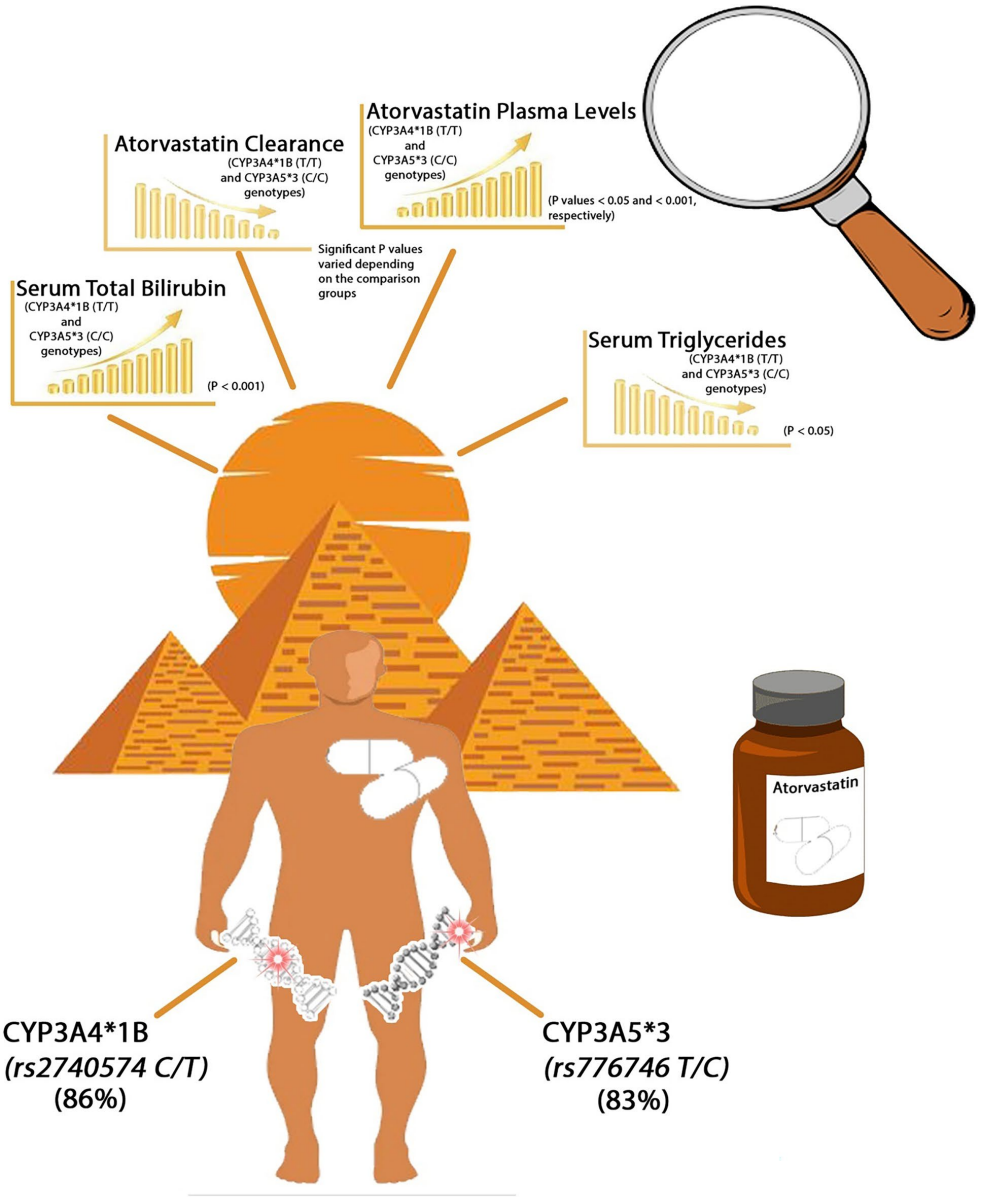

## Introduction

Atorvastatin is regarded as one of the most frequently recommended medications and statins most often used globally [1]. It is currently recommended for the management of dyslipidemia and hypercholesterolemia [2]. With respect to managing hyperlipidemia, statins are usually the preferred medication prescribed by physicians as the first course of treatment [3]. However, responses to statin therapy exhibit evident interpersonal deviations in the expected lipid-lowering efficacy [3], where atorvastatin is an example: polymorphisms in genes responsible for metabolism, distribution, and uptake might modulate therapeutic outcomes [3]. These deviations in responses are a major clinical problem [4, 5].

The enzymes mediate the metabolism of atorvastatin, *CYP3A4*, and *CYP3A5* [6]; consequently, genetic variations in *CYP3A4* or *CYP3A5* lead to dissimilarities in the *CYP3A* metabolic pathways of atorvastatin [6]. The CYP3A4*1B SNP is linked to decreased enzymatic activity [3]. Nevertheless, there have been conflicting reports regarding its relationship with atorvastatin [3, 7–12]. Similarly, the CYP3A5*3 SNP leads to a truncated malfunctioning protein in homozygous cases (nonexpressors) [13]. However, its association with atorvastatin has been the subject of contradictory reports [7, 9, 10, 14–18].

SNPs are the most basic type of DNA variation found in individuals [19]. They are variations in the DNA sequence that occur when a single nucleotide in the genome is different in paired chromosomes [20]. The *CYP3A4* gene is located on chromosome 7, and CYP3A4*1B (*rs2740574*) is a SNP in which a C allele is substituted with a T allele at chromosome 7:99,784,473 [21, 22]. Additionally, the *CYP3A5* gene is located on chromosome 7, and CYP3A5*3 (*rs776746*) is a SNP in which a T allele is substituted with a C allele at chromosome 7:99,672,916 [21, 23]. The CYP3A4*1B variant has been linked to obesity, nonalcoholic fatty liver disease (NAFLD), prostate cancer, and premature onset of menstruation, a well-established risk factor for the development of breast cancer [24–26]. Notably, the CYP3A5*3 SNP has been associated with the likelihood of developing chronic myeloid leukemia (CML) [27]. Moreover, the CYP3A5*3 variant has been associated with a greater risk of hypertension and increased serum TG (a type of fat that increases the risk of cardiovascular diseases (CVDs)) [28, 29]. Furthermore, both SNPs, CYP3A4*1B and CYP3A5*3, decrease the metabolic activities of *CYP3A4* and *CYP3A5*, respectively [3, 13]. A decrease in *CYP3A* activity was associated with elevated serum levels of TB (which includes both direct and indirect bilirubin) and alanine aminotransferase (ALT) [30].

A recent World Health Organization (WHO) report stated that almost half of all deaths in Egypt are due to CVDs [31, 32]. Dyslipidemia increases the risk of CVDs [32–34]. Several studies have shown that 37% of Egyptians experience hyperlipidemia [32–34]. The most commonly prescribed lipid-lowering drug therapy in Egypt is statin monotherapy, with atorvastatin being the most frequently recommended statin [35]. To the best of our knowledge, the effects of the CYP3A4*1B (*rs2740574* C/T) or CYP3A5*3 (*rs776746* T/C) polymorphisms on atorvastatin efficacy and safety in Egyptians have not been previously studied.

Numerous studies have evaluated the impact of *CYP3A4* and *CYP3A5* genetic polymorphisms in response to atorvastatin therapy; however, their findings are inconsistent or discordant [3, 7–12, 14–18]. Accordingly, the aim of this study was to explore the potential influence of CYP3A4*1B/CYP3A5*3 variants on atorvastatin treatment in the Egyptian population. This study aimed to determine the allelic frequencies of the CYP3A4*1B and CYP3A5*3 SNPs among Egyptian participants. This study specifically focused on changes in serum lipid and lipoprotein levels, liver enzymes, TB, and CK after four weeks of treatment with 40 mg atorvastatin. Moreover, researchers intend to assess posttreatment atorvastatin plasma levels via liquid chromatography‒tandem mass spectrometry (LC‒MS/MS). Furthermore, this study evaluated the pharmacokinetics of atorvastatin in individuals carrying the CYP3A4*1B (T/T) and CYP3A5*3 (C/C) genotypes.

## Materials and methods

**Fig. 1:**
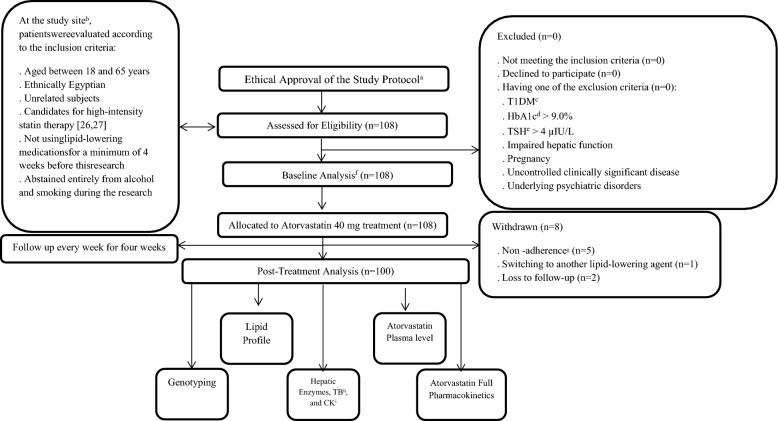
Research methodology flow chart. **a** Ethical approval: The study protocol and informed consent were approved by the Egyptian Russian University (ERU), Cairo, Egypt, in addition to Badr Hospital, Cairo, Egypt, and Universiti Sains Malaysia (USM), Penang, Malaysia; **b** Study site: the endocrinology clinic, Badr Hospital, Helwan University, Cairo, Egypt; **c** T1DM: type 1 diabetes mellitus (insulin deficiency caused by the loss of pancreatic β-cells results in hyperglycemia, a long-lasting illness) [38].), **d** HbA1c: glycated hemoglobin (glycemia represents glycemic control over a prolonged period [39]. The American Diabetes Association advised individuals diagnosed with type 2 diabetes and HbA1c levels exceeding 9.0% to consider the use of insulin [40].), **e** TSH: thyroid stimulating hormone (which is generated by the anterior pituitary gland and serves as the primary stimulant for the thyroid gland's production of thyroid hormones [41].), **f** Baseline analysis: baseline lipid profile, hepatic enzymes, TB^g^, and CK^h^ analysis (n = 108), **g** Nonadherence occurred if a participant did not take 40 mg atorvastatin tablet as prescribed, **h** TB: total bilirubin (TB measurement includes direct and indirect bilirubin levels.), **i** CK: creatine kinase (It facilitates energy reactions in muscle cells. Elevated levels of CK typically occur after strenuous and prolonged exercise and eccentric muscle training [42].)

This prospective cohort study enrolled subjects at baseline before 40 mg atorvastatin treatment. The participants were followed up until the fourth week after atorvastatin administration (Fig. 1).

### Reagents

#### Reagents for genotyping

A DNA purification mini kit, the QIAamp DNA Mini Kit, was obtained from Qiagen, Hilden, Germany. This kit comprises lysis (AL), washing (AW1 and AW2), and elution (AE) buffers. In addition, the kits used included QIAGEN Protease, Protease Solvent, Proteinase K, collection tubes (2 ml), and spin columns. The TaqMan master mix for genotyping was supplied by Applied Biosystems, California, USA. The AmpliTaq Gold DNA Polymerase UP (Ultra-Pure), Passive Reference 1, dNTPs without dUTP, and optimized mix components were included in the master mix. Additionally, the genotyping assay kits used for the CYP3A4*1B (*rs2740574* C/T) and CYP3A5*3 (*rs776746* T/C) SNPs were obtained from Thermo Fisher Scientific (Massachusetts, USA). Each kit contained reverse and forward primers that were appropriate for sequencing and two TaqMan-MGB probes for recognizing SNPs. One probe was a dye-labeled VIC reporter (wild-type allele), and the other was a dye-labeled FAM reporter (variant allele). Furthermore, the context sequence for *rs2740574* is [VIC/FAM]: TAAAATC TATTAAATCGCCTCTCTC[C/T]TGCCCTTGTCTCTATGGCTGTCCTC. For *rs776746,* the context sequence is [VIC/FAM]: ATGTGGTCCAAACAGGGAAGAGATA[T/C]TGAAAGAC AAAAGAGCTCTTTAAAG.

#### Reagents for LC‒MS/MS analysis

Atorvastatin was a generous gift from EIPICO, Al Sharkia, Egypt, whereas rosuvastatin (internal standard [IS]) was a lavish gift from Mash Premiere, Cairo, Egypt. Sigma‒Aldrich Corp., Missouri, USA, provided LC‒MS grade water, acetonitrile, formic acid (~ 98%), ammonium formate (≥ 99.0%), and methanol. Blank human plasma was obtained from the local government blood bank in Al Sharkia, Egypt.

### Study population

#### Ethical considerations

The research protocol and informed consent form (ICF) were approved by the Scientific Research Ethics Committee, Faculty of Pharmacy, Egyptian Russian University (ERU), Cairo, Egypt (code: ECH-022-May 2022). In addition, the Badr Hospital Research Ethics Committee, Helwan University, Cairo, Egypt, approved the study in October 2022. Additionally, Jawatankuasa Etika Penyelidikan Manusia (JEPeM), Universiti Sains Malaysia (USM), and Penang, Malaysia, approved the research in February 2023 (code: USM/JEPeM/22090641). This work complied with the World Medical Association's Code of Ethics (Declaration of Helsinki). The patient's participation was voluntary, and he or she had the right to withdraw his or her consent or to terminate contributions at any time without consequence or loss of benefits. No one can view the patient’s medical information except for the research team (principal investigator and coresearchers). Data encryption and coding are limited to only the research team. Moreover, individual privacy will be maintained in all published and written data from the study.

#### Subject criteria

Candidates for high-intensity statin therapy [36, 37] who provided voluntary ICF were enrolled in this study and were selected from the endocrinology clinic at Badr Hospital, Helwan University, Cairo, Egypt. All the participants (n = 108) were ethnically Egyptian, unrelated, and aged between 18 and 65 years. None of the participants had used lipid-lowering agents for at least four weeks before this study. All participants abstained entirely from alcohol and smoking during the study. They were also instructed to refrain from participating in intense physical exertion starting one week before and throughout the study. Subjects with type 1 diabetes mellitus (DM) or a glycated hemoglobin (HbA1c) level > 9.0% were not included. Additionally, individuals with uncontrolled hypothyroidism (thyroid stimulating hormone (TSH) > 4 µIU/L) and patients whose liver function was poor (aspartate aminotransferase (AST) or ALT > 1.4 ULN) were excluded. In addition, pregnant individuals who had uncontrolled clinically significant disease or underlying psychiatric disorders were also excluded. Owing to patients’ nonadherence to 40 mg atorvastatin (n = 5), switching to another lipid-lowering agent (n = 1), or failure to follow up (n = 2), eight subjects were withdrawn from the study (Fig. 1).

### Genotyping of the CYP3A4*1B and CYP3A5*3 polymorphisms

Determination of CYP3A4*1B (*rs2740574* C/T) and CYP3A5*3 (*rs776746* T/C) gene polymorphisms was performed via real-time quantitative polymerase chain reaction (qPCR).

#### Sampling

Venous blood (2 mL) from all the participants was collected into a tri-potassium EDTA vacutainer, and the tube was inverted several times and inspected to exclude the possibility of clots. Prior to DNA extraction, the samples were stored at − 80 °C. The samples were assayed via qPCR through two steps: genomic DNA extraction and amplification/real-time PCR allelic discrimination assays.

#### Genomic DNA extraction

The DNA extraction method relied on the QIAamp DNA Mini Kit (QIAGEN, Aarhus, Denmark), which followed the manufacturer's instructions.

#### Amplification and real-time PCR allelic discrimination assays

The extracted DNA was subjected to amplification and allelic discrimination via real-time PCR with sequence-specific primers. In each run, the required number of PCR tubes was calculated. Additionally, 2 μL of extracted DNA and 10 μL of TaqMan Universal PCR Master Mix were added to each tube. Next, one microliter of the 20 × working stock of the SNP genotyping assay mixture and seven microliters of DNase-free water were added. Next, the samples were transferred to a thermal cycler as Applied Biosystems, California, USA specified. For standard PCR runs, Applied Biosystems recommends a 10-min pre-PCR activation step at 95 °C, followed by 40 cycles of denaturation (15 s at 95 °C) and annealing/extension (60 s at 60 °C).

### Atorvastatin effectiveness and safety

All participants were screened for serum lipid profiles (HDL-C, LDL-C, TC, and TG), ALT, AST, TB, and CK at baseline and after atorvastatin 40 mg for four weeks.

### Atorvastatin plasma level

After four weeks of atorvastatin treatment and at equal intervals before subsequent dosing, a 1 mL blood sample was withdrawn from each patient into a tri-potassium EDTA vacutainer. The plasma samples were separated immediately, and before LC‒MS/MS analysis, each sample was preserved at − 80 °C.

#### Chromatographic conditions

Chromatographic analysis was performed via Agilent 1260 Infinity Quaternary Liquid Chromatography (Agilent Technologies, Waldbronn, Germany). Atorvastatin and the IS were separated on an ACQUITY UPLC BEH C18 column (1.7 µm, 2.1 mm × 100 mm) (Waters, Massachusetts, USA). The mobile phases included ammonium formate (10 mM) and formic acid (0.04%) in water as mobile phase A, in addition to acetonitrile as mobile phase B. For separation, gradient elution was applied following the following procedure: 0–2 min, 30% B; 2–3 min, 30–60% B; 3–4 min, 60–80% B; 4–5 min, 80–95% B; 5–6 min, 95% B; 6–7 min, 95–80% B; 7–8 min, 80% B; 8–9 min, 80–60% B; and 9–10 min, 60–30% B. Ten minutes was the run time. Elution was carried out with a 5 µL injection volume, a 20 °C sample temperature, and a 40 °C column temperature. A flow rate of 0.2 ml/min was utilized.

#### Mass spectrometry conditions

An Agilent 6460 Triple Quad mass spectrometer (Model K6460) (Agilent Technologies, California, USA) was used, and LC‒MS/MS analysis was conducted. The concentrations of atorvastatin and the IS in the plasma samples were ascertained via an electrospray ionization (ESI) source operating in positive ion mode for detection via mass spectrometry. Multiple reaction monitoring (MRM) mode was used to identify transition ions and quantify the most appropriate mass transitions. For atorvastatin, quantification was performed via the transitions of m/z 559.2 → 440.3 and m/z 482.3 → 258.1 for the IS. The mass spectrometer's primary operating parameters were as follows: ion spray voltage, 5.5 kV; ion source temperature, 500 °C; ion source gas1, 20 psi; gas2, 20 psi; collision gas, 30 psi; and curtain gas, 20 psi. Agilent MassHunter Workstation Software (B.08.00, Agilent Technologies, California, USA) was used to acquire the data.

#### Preparation of calibration standards and quality control samples

The analyte and IS standard stock solutions were made independently, with a 500 µg/ml concentration in methanol. A -20 °C temperature was used for storing stock solutions until they were used to create working solutions. After the above stock solutions were diluted in pure methanol, various working standard solutions of atorvastatin (10–500 ng/mL) and the IS (500 ng/mL) were created and stored at − 20 °C. To produce 1–1000 ng/ml calibration standards, the blank plasma samples were spiked with these standard working solutions. Additionally, QC samples were produced with 50 ng/mL IS and low (LQC), medium (MQC), and high (HQC) quality control doses of atorvastatin (2, 500, and 750 ng/ml, respectively).

#### Plasma sample preparation

Six replicates of the plasma calibration standards with concentrations ranging from 1 to 1000 ng/mL were prepared in 1.8 mL Eppendorf tubes. To create these standards, 20 µL of internal standards in addition to suitable aliquots of atorvastatin working solutions were added to 200 µL of blank human plasma. The generated plasma samples were precipitated with acetonitrile (1 mL). After vortexing at high speed for one minute, the mixture was spun in a centrifuge for 15 min at a speed of 20,000 rpm. After being transferred to a new vial, the supernatant was allowed to evaporate at room temperature and then dried with a nitrogen spray. For LC‒MS/MS analysis, a total of one hundred µl of water/acetonitrile with a ratio of 70:30 (v/v) was used for reconstitution. The injection volume was 5 µL.

### Atorvastatin full pharmacokinetic profile

Blood samples were drawn from five subjects who carried both the homozygous mutant genotypes (T/T) and (C/C) of the SNPs CYP3A4*1B and CYP3A5*3, respectively. The following samples were collected: 0.5 ml at predose, 0.5 ml at two hours, 0.5 ml at twelve hours, and 0.5 ml at eighteen hours postdose. The plasma levels of atorvastatin at the four time points were assessed via LC‒MS/MS analysis.

### Data analysis

The collected data were revised, coded, tabulated, and introduced to a PC via the Statistical Package for Social Science (IBM Corp. Released 2017. IBM SPSS Statistics for Windows, Version 26.0. Armonk, NY: IBM Corp). The quantitative variables were normally distributed. Student’s t test was used to compare quantitative variables between two study groups. One‐way analysis of variance (ANOVA) was used to compare quantitative variables between more than two study groups, with Tukey's post hoc test for pairwise comparisons. Pearson's correlation was used to determine whether there was a linear relationship between two variables in the case of normally distributed continuous variables. In addition, Kendall's tau-b analysis was used to determine correlations for nonnormal distributions. Linear regression analysis was used to predict a continuous outcome. Logistic regression analysis was used to predict a categorical outcome (binary). Receiver operating characteristic (ROC) curves were used to assess the performance of the prediction model for discrimination. A P value < 0.05 was considered statistically significant.

The pharmacokinetic profile of atorvastatin was calculated via linear regression via the equation y = a + bx, where y is the area under the peak (AUP) ratio of the drug to the internal standard, (a) is the intercept, (b) is the slope and (x) is the concentration of atorvastatin. The relative standard deviation (RSD) was calculated for all values. Intraday and interday accuracy, precision, extraction recovery, and matrix effect results were compared at each QC concentration level via Student’s t test. Pharmacokinetic parameters were estimated via model‐independent methods (Gibaldi, M. and Perrier, D. approach, 1982) [43, 44]. The terminal elimination rate constant (k) was estimated via linear regression analysis of the terminal portion of a drug’s ln–linear blood concentration–time profile. The terminal elimination half‐life (t_1/2_) was calculated from the terminal elimination rate constant via the formula t_1__/2_ = 0.693/k. The linear trapezoidal rule was used to calculate the area under each drug concentration‒time curve (AUC_0–τ_, µg h/L) from dosing to the end of the dosing interval (τ = 24 h). The apparent oral clearance (Cl/F) was calculated from the dose/AUC_0–τ_. Student’s t‐test was used to examine the concentration difference each day, and ANOVA was used to evaluate the reproducibility of the assay. The level of confidence was 95%.

## Results

### Subjects’ demographics

The demographic and clinical characteristics of the remaining patients are shown in Table 1. All the subjects recruited in this study were Egyptians. The patients were between 20 and 65 years old. The subjects were primarily females (63%), whereas males represented 37% of the total patients. Most of the participants were obese (90%), had class I obesity (32%), had class II obesity (41%), or had class III obesity (17%). Furthermore, the majority of the patients were diabetic (85%), hypertensive (59%), or nonsmokers (73%). All the patients were candidates for high-intensity statin therapy. The participants were either predisposed to or already had atherosclerotic cardiovascular disease (ASCVD). Approximately two-thirds (62%) of the subjects were on low-dose aspirin as a prophylactic therapy for CVD events. None of the patients were on antihyperlipidemic agents for at least four weeks before the study.

**Table 1:** Demographic and clinical characteristics of the study subjects (n = 100)

Variable		n (%)
Age, mean (SD), year^a^		54.25 (9.21)
Gender	Male	37 (37%)
	Female	63 (63%)
BMI classification	Normal	1 (1%)
	Overweight	9 (9%)
	Obese Class I	32 (32%)
	Obese Class II	41 (41%)
	Obese Class III	17 (17%)
Smoking	Never	73 (73%)
	Former	10 (10%)
	Current	17 (17%)
History of diabetes	Yes	85 (85%)
	No	15 (15%)
On hypertension treatment	Yes	59 (59%)
	No	41 (41%)
On Aspirin	Yes	62 (62%)
	No	38 (38%)

^a^Data for all continuous variables are expressed as the mean (standard deviation (SD))

### Allele frequencies of the CYP3A4*1B (*rs2740574 *C/T) and CYP3A5*3 (*rs776746* T/C) SNPs among the study population in Egypt

#### CYP3A4*1B SNP

The genotyping results are shown in Table 2. Homozygosity (T/T) of the CYP3A4*1B (*rs2740574* C/T) SNP was prevalent among the study participants (74%), whereas heterozygosity (C/T) was represented by approximately one-quarter (24%) of the patients. In addition, this study demonstrated that the frequency of the wild (C/C) genotype among Egyptians was noticeably low (2%). The frequency of the CYP3A4*1B variant allele was highly predominant at 86%.

**Table 2:** Allelic and genotype frequencies and percentages of the CYP3A4*1B (*rs2740574* C/T) and CYP3A5*3 (*rs776746* T/C) polymorphisms

All Subjects (n = 100)	n	%^*^
A. CYP3A4*1B (*rs2740574* C/T) genotype		
(CC) genotype	2	2
(CT) genotype	24	24
(TT) genotype	74	74
Allele frequency		
C (Wild)	28	14
T	172	86
B. CYP3A5*3 (*rs776746* T/C) genotype		
(TT) genotype	1	1
(TC) genotype	32	32
(CC) genotype	67	67
Allele frequency		
T (Wild)	34	17
C	166	83

^*^(%): The percentage is determined by dividing the frequency in each category by the total number of participants and then multiplying the result by 100%

Kendall's tau-b analysis revealed statistically significant correlations (P values < 0.001) between the three genotypes of CYP3A4*1B (*rs2740574* C/T) and the three genotypes of CYP3A5*3 (*rs776746* T/C) (Table 3).

**Table 3:** Significant correlations between the CYP3A4*1B (rs2740574 C/T)/CYP3A5*3 (rs776746 T/C) genotypes and other parameters under investigation among the study participants (n = 100)

Genotype		Variable	r^a^	P value	Correlation Coefficient Type
CYP3A4*1B (rs2740574 C/T)	(C/C) genotype n = 2	TG^b^ % CHANGE	0.30	0.003	Pearson’s
		CK^c^ CHANGE	− 0.60	< 0.001	Pearson’s
		CYP3A5*3 (T/T) genotype	0.70	< 0.001	Kendall's tau-b
	(C/T) genotype n = 24	TB^d^ % CHANGE	0.30	0.004	Pearson’s
		Atorvastatin plasma level	− 0.60	< 0.001	Pearson’s
		CYP3A5*3 (T/C) genotype	0.50	< 0.001	Kendall's tau-b
	(T/T) genotype n = 74	TB^d^ % CHANGE	− 0.30	0.006	Pearson’s
		CK^c^ CHANGE	0.30	0.009	Pearson’s
		Atorvastatin plasma level	0.62	< 0.001	Pearson’s
		CYP3A5*3 (C/C) genotype	0.51	< 0.001	Kendall's tau-b
CYP3A5*3 (rs776746 T/C)	(T/T) genotype n = 1	CYP3A4*1B (C/C) genotype	0.70	< 0.001	Kendall's tau-b
	(T/C) genotype n = 32	Atorvastatin plasma level	− 0.73	< 0.001	Pearson’s
		CYP3A4*1B (C/T) genotype	0.50	< 0.001	Kendall's tau-b
	(C/C) genotype n = 67	Atorvastatin plasma level	0.80	< 0.001	Pearson’s
		CYP3A4*1B (T/T) genotype	0.51	< 0.001	Kendall's tau-b

^a^(r): Correlation coefficient, ^b^(TG): triglycerides, ^c^(CK): creatine kinase, ^d^(TB): total bilirubin

#### CYP3A5*3 SNP

The frequency of homozygotes (C/C) of the CYP3A5*3 (*rs776746* T/C) variant was high. This genotype was found in approximately two-thirds (67%) of the study participants, whereas approximately one-third (32%) of the participants were heterozygotes (T/C) of CYP3A5*3. With respect to the CYP3A5*3 SNP, this study revealed that the prevalence of the wild-type (T/T) genotype was significantly lower (1%) in Egyptian study subjects. The allelic frequency of the CYP3A5*3 variant was highly prevalent at 83% (Table 2).

Kendall's tau-b analysis revealed significant correlations (P values < 0.001) between the CYP3A5*3 (*rs776746* T/C) genotype and the CYP3A4*1B (*rs2740574* C/T) genotype (Table 3).

#### Genotype and prediction statistical analysis

Logistic regression as an extrapolation model revealed that both the CYP3A4*1B (*rs2740574* C/T) genotype (C/T) and (T/T) were predictors of the CYP3A5*3 (*rs776746* T/C) genotype (T/C) and (C/C), respectively. Logistic regression revealed a significant relationship between the CYP3A5*3 (T/C) genotype as the dependent binary variable and the CYP3A4*1B (C/T) genotype as the predictor variable. The odds ratio (OR) was 9.88, 95% confidence interval (CI): 3.5–28.1, P < 0.001). Logistic regression analysis revealed a significant relationship between the CYP3A5*3 (C/C) genotype, the dependent binary variable, and the CYP3A4*1B (T/T) genotype, the independent variable. The OR was 11.63 (95% CI: 4.1–33.0, P < 0.001) (Table 4).

**Table 4:** Relationships between genotypes of CYP3A4*1B (rs2740574 C/T) and CYP3A5*3 (rs776746 T/C) and other investigated parameters among the study subjects (n = 100)

A. Linear regression				
Independent variable (Predictor)	Dependent variable (Outcome)	R^2a^	β^b^	P value
CYP3A4*1B (C/T) genotype	Atorvastatin plasma level (ng/ml)	0.3	− 4.08	< 0.001
CYP3A4*1B (T/T) genotype		0.4	4.22	< 0.001
CYP3A5*3 (T/C) genotype		0.5	− 4.68	< 0.001
CYP3A5*3 (C/C) genotype		0.6	4.79	< 0.001
CYP3A4*1B (C/C) genotype	Change in CK^c^, (U/L)	0.3	− 84.40	< 0.001

B. Logistic regression					
Independent variable (predictor)	Dependent variable (outcome)	OR^d^	95% CI^e^	β^b^	P value
Atorvastatin plasma level	CYP3A4*1B (T/T) genotype	1.96	(1.5–2.6)	0.67	< 0.001
	CYP3A5*3 (C/C) genotype	2.43	(1.8–3.3)	0.89	< 0.001
CYP3A4*1B (C/T) genotype	CYP3A5*3 (T/C) genotype	9.88	(3.5–28.1)	2.29	< 0.001
CYP3A4*1B (T/T) genotype	CYP3A5*3 (C/C) genotype	11.63	(4.1–33.0)	2.45	< 0.001

^a^R^2^: coefficient of determination, ^b^β: regression coefficient, ^c^CK: creatine kinase, ^d^OR: odds ratio, ^e^95% CI: 95% confidence interval

### Effect of genetic polymorphisms on atorvastatin effectiveness

#### The CYP3A4*1B (*rs2740574* C/T) genetic variant

The associations between the CYP3A4*1B (*rs2740574* C/T) variant and serum lipid or lipoprotein levels were analyzed. Data from patients carrying the wild-type (C/C) genotype were compared with those from patients carrying the (T/T) and (C/T) genotypes. In addition, data from subjects carrying the homozygous (T/T) genotype were compared with those from subjects heterozygous (C/T) for the CYP3A4*1B (*rs2740574* C/T) SNP (Table 5). ANOVA revealed no evidence of a difference in baseline serum lipid or lipoprotein levels among the three genotype carriers of the CYP3A4*1B variant (Table 5).

**Table 5:** Association between CYP3A4*1B/CYP3A5*3 genetic polymorphisms, serum lipid/lipoprotein levels, and other clinical parameters before and after four weeks of treatment with atorvastatin

Variable		All Subjects Mean (SD)^a^ (n = 100)	CYP3A4*1B (rs2740574 C/T) Mean (SD)^a^ (n = 100)			P Value (ANOVA^b^)	P Value (ANOVA^b^ Post hoc^c^)			CYP3A5*3 (rs776746 T/C) Mean (SD)^a^ (n = 99)		P Value (Student's t test)
			CC (n = 2)	CT (n = 24)	TT (n = 74)		*C/*C vs *C/*T	*C/*C vs. *T/*T	* C/*T vs. *T/*T	CC (n = 67)	TC (n = 32)	
BMI^d^, kg/m^2^		35.70 (4.38)	30.50 (0.71)	35.34 (4.78)	35.96 (4.24)	0.20	0.29	0.19	0.82	35.36 (4.11)	36.60 (4.82)	0.19
Systolic blood pressure, mmHg		132.44 (11.00)	133.50 (9.19)	132.79 (8)	132.30 (11.94)	0.97	0.99	0.99	0.98	131.90 (11.96)	133.75 (8.85)	0.44
Diastolic blood pressure, mmHg		81.49 (6.78)	79.00 (8.49)	82.67 (5.11)	81.18 (7.24)	0.57	0.75	0.90	0.62	81.54 (7.15)	81.66 (5.97)	0.94
Atorvastatin level, ng/ml		6.49 (3.00)	3.08 (0.82)	3.39 (1.03)	7.59 (2.69)	< 0.001*	0.98	0.03*	< 0.001*	8.07 (2.33)	3.30 (1.00)	< 0.001*
TC^e^, mg/dL	Pretreatment	240.48 (48.82)	223.6 (8.77)	244.25 (45.54)	239.72 (50.64)	0.82	0.84	0.89	0.92	241.19 (48.54)	(239.73) 50.76	0.89
	Posttreatment	149.10 (39.24)	115.45 (6.43)	149.29 (32.91)	149.95 (41.41)	0.48	0.48	0.44	0.99	150.32 (39.82)	147.75 (38.60)	0.76
	CHANGE^m^	− 91.38 (25.67)	− 108.15 (2.33)	− 94.96 (22.48)	− 89.77 (26.82)	0.45	0.77	0.58	0.67	− 90.87 (27.68)	− 91.98 (21.56)	0.84
	% CHANGE^n^	− 38.20 (8.06)	− 48.38 (0.85)	− 38 (6.1)	− 37.7 (8.6)	0.16	0.25	0.15	0.77	− 37.81 (8.85)	− 38.69 (6.04)	0.61
LDL-C^f^, mg/dL	Pretreatment	172.33 (46.11)	183.65 (11.81)	177.97 (46.52)	170.20 (46.70)	0.73	0.99	0.91	0.76	171.21 (46.59)	174.60 (46.47)	0.74
	Posttreatment	87.14 (35.24)	74.85 (4.45)	88.14 (32.09)	87.15 (36.81)	0.88	0.87	0.88	0.99	87.31 (36.02)	87.27 (34.57)	1.00
	CHANGE^m^	− 85.19 (25.03)	− 108.80 (7.35)	− 89.82 (21.49)	− 83.05 (26.00)	0.21	0.56	0.32	0.48	− 83.90 (26.90)	− 87.34 (20.99)	0.53
	% CHANGE^n^	− 50.42 (10.84)	− 59.24 (.20)	− 51.60 (8.16)	− 49.80 (11.63)	0.40	0.61	0.45	0.76	− 49.82 (11.73)	51.38 (8.84)	0.51
HDL-C^g^, mg/dL	Pretreatment	33.91 (9.71)	20.00 (0.00)	35.09 (11.28)	33.91 (9.06)	0.11	0.09	0.11	0.86	34.30 (9.16)	33.55 (10.76)	0.72
	Posttreatment	37.31 (10.61)	22.00 (0.00)	38.39 (12.14)	37.37 (9.98)	0.11	0.09	0.11	0.91	37.76 (10.09)	36.83 (11.62)	0.69
	CHANGE^m^	3.39 (2.84)	2.00 (0.00)	3.30 (1.06)	3.46 (3.25)	0.76	0.81	0.76	0.97	3.46 (3.40)	3.28 (1.05)	0.77
	% CHANGE^n^	10.40 (8.55)	10.00 (0.00)	9.57 (1.64)	10.68 (9.90)	0.86	0.99	0.99	0.85	10.62 (10.40)	9.94 (1.70)	0.72
TG^h^, mg/dL	Pretreatment	171.19 (79.70)	99.65 (15.06)	155.98 (48.68)	178.05 (87.18)	0.22	0.60	0.36	0.47	178.46 (89.82)	157.88 (51.89)	0.15
	Posttreatment	124.93 (58.71)	93.00 (9.90)	115.90 (36.27)	128.72 (64.73)	0.48	0.86	0.68	0.62	127.93 (65.41)	119.87 (42.48)	0.53
	CHANGE^m^	− 46.26 (37.34)	− 6.65 (24.96)	− 40.10 (17.60)	− 49.33 (41.47)	0.18	0.44	0.25	0.54	− 50.53 (43.61)	− 38.01 (16.64)	0.04*
	% CHANGE^n^	− 25.95 (10.39)	− 4.84 (24.32)	− 25.51 (8.35)	− 26.70 (10.17)	0.01*	0.02*	0.01*	0.88	− 26.96 (10.90)	− 23.96 (9.21)	0.18
ALT^i^, U/L	Pretreatment	18.64 (4.80)	13.50 (2.12)	18.00 (4.91)	18.99 (4.76)	0.21	0.41	0.25	0.66	19.48 (4.96)	17.09 (4.00)	0.02*
	Posttreatment	23.54 (10.75)	13.00 (4.24)	21.42 (9.02)	24.51 (11.20)	0.18	0.53	0.29	0.44	24.51 (8.57)	21.94 (14.22)	0.27
	CHANGE^m^	4.90 (8.83)	− 0.50 (2.12)	3.42 (5.92)	5.53 (9.63)	0.41	0.82	0.61	0.57	5.03 (6.23)	4.84 (12.84)	0.92
	% CHANGE^n^	25.41 (50.86)	− 5.00 (16.50)	16.24 (31.33)	29.21 (55.92)	0.39	0.84	0.62	0.53	25.95 (34.80)	25.60 (74.99)	0.97
AST^j^, U/L	Pretreatment	20.22 (7.13)	17.00 (1.41)	18.00 (5.42)	21.03 (7.55)	0.16	0.98	0.71	0.17	21.51 (7.93)	17.66 (4.19)	< 0.01*
	Posttreatment	26.77 (13.18)	16.50 (2.12)	23.42 (8.74)	28.14 (14.25)	0.17	0.75	0.43	0.28	27.63 (11.88)	25.34 (15.69)	0.42
	CHANGE^m^	6.55 (10.28)	− 0.50 (0.71)	5.42 (6.18)	7.11 (11.37)	0.49	0.72	0.56	0.77	6.12 (7.87)	7.69 (14.22)	0.48
	% CHANGE^n^	32.45 (48.75)	− 3.13 (4.42)	30.82 (32.70)	33.95 (53.36)	0.56	0.62	0.54	0.96	28.81 (33.63)	41.29 (70.87)	0.24
TB^k^, mg/dL	Pretreatment	0.69 (0.14)	0.65 (0.07)	0.63 (0.11)	0.72 (0.15)	0.02*	0.97	0.79	0.02*	0.72 (0.14)	0.63 (0.12)	< 0.01*
	Posttreatment	0.87 (0.22)	0.81 (0.13)	0.86 (0.20)	0.87 (0.23)	0.90	0.94	0.91	0.98	0.89 (0.19)	0.82 (0.28)	0.15
	CHANGE^m^	0.17 (0.17)	0.16 (0.06)	0.23 (0.15)	0.16 (0.17)	0.14	0.80	1.00	0.12	0.17 (0.13)	0.19 (0.23)	0.50
	% CHANGE^n^	26.26 (23.73)	23.45 (7.24)	38.26 (22.79)	22.44 (23.17)	0.02*	0.66	1.00	0.01*	24.10 (19.28)	31.03 (31.16)	0.25
CK^l^, U/L	Pretreatment	73.79 (31.85)	159.00 (110.31)	64.96 (28.36)	74.35 (27.03)	< 0.001*	< 0.001*	< 0.001*	0.37	74.01 (27.54)	73.09 (40.29)	0.89
	Posttreatment	84.50 (33.51)	87.00 (8.49)	72.42 (33.30)	88.35 (33.31)	0.13	0.82	1.00	0.11	87.57 (33.25)	77.81 (34.12)	0.18
	CHANGE^m^	10.71 (21.35)	− 72.00 (118.79)	7.46 (12.30)	14.00 (13.24)	< 0.001*	< 0.001*	< 0.001*	0.26	13.55 (12.33)	4.72 (32.83)	0.06
	% CHANGE^n^	16.33 (20.86)	− 25.50 (57.02)	12.32 (18.68)	18.77 (19.39)	0.01*	0.03*	0.01*	0.36	18.42 (18.65)	12.01 (24.89)	0.16

^a^Values are given as the mean (standard deviation (SD)), ^b^ANOVA: analysis of variance, ^c^Post hoc: Tukey HSD test, ^d^BMI: body mass index, ^e^TC: total cholesterol, ^f^LDL-C: low-density lipoprotein cholesterol, ^g^HDLC: high-density lipoprotein cholesterol, ^h^TG: triglycerides, ^i^ALT: alanine amino transferase, ^j^AST: aspartate amino transferase, ^k^TB: total bilirubin, ^l^CK: creatine kinase, ^m^CHANGE: change from baseline. The calculation formula for changes in laboratory parameters was as follows: (a parameter posttreatment minus the same parameter pretreatment), n. % CHANGE: percentage change from baseline. The calculation formula for % changes in laboratory parameters was as follows: (a parameter posttreatment minus the same parameter pretreatment, then divided by the parameter pretreatment, and multiplied by 100)

^*^Significant p value

##### Serum TG percentage reduction

The percentage reduction in the serum TG concentration was lower in the CYP3A4*1B (C/C) (wild genotype) group than in the C/T and T/T genotype groups (P value < 0.05). The percentage reduction in serum TG was 4.84 ± 24.32 in the CYP3A4*1B (C/C) genotype group (Table 5). Among the CYP3A4*1B (C/T) and (T/T) genotype carriers, the serum TG percentage reductions were 25.51 ± 8.35% and 26.70 ± 10.17%, respectively (Table 5).

Pearson’s correlation revealed a weak positive correlation between TG percentage reduction and the CYP3A4*1B (C/C) genotype ((r = 0.30), (n = 2), and (P value < 0.05)) (Table 3).

#### The CYP3A5*3 (*rs776746* T/C) genetic variant

The relationships between the CYP3A5*3 (*rs776746* T/C) variant and blood lipid or lipoprotein levels were investigated. Data from patients carrying the homozygous mutant genotype (C/C) were compared with those from patients carrying the heterozygous genotype (T/C) (Table 5). The genotyping findings revealed that only one patient carried the wild-type (T/T) genotype. Thus, data regarding this homozygous wild-type genotype were excluded from the statistical analysis.

There was no evidence of a difference between the two genotype carriers ((C/C) or (T/C) of the variant CYP3A5*3) regarding the baseline serum levels of lipids or lipoproteins (Table 5).

##### Serum TG reduction

The serum TG concentration was greater in the C/C genotype carriers (50.53 ± 43.61) than in the C/T genotype carriers (38.01 ± 16.64) (P value < 0.05) (Table 5).

### Effect of genetic polymorphisms on the safety of atorvastatin

#### The CYP3A4*1B (*rs2740574* C/T) genetic variant

The associations between the CYP3A4*1B (*rs2740574* C/T) variant and serum ALT, AST, TB, and CK levels were studied.

##### Serum ALT/AST

There was no evidence of a difference between the three CYP3A4*1B genotypes regarding the baseline and posttreatment serum ALT and AST levels (P values > 0.05) (Table 5).

##### Serum TB

Baseline serum TB: Compared with carriers of the C/T genotype, carriers of the CYP3A4*1B (T/T) genotype had greater baseline TB (mg/dL) (P value < 0.05). The baseline TB level (mg/dL) in the case of the CYP3A4*1B (T/T) genotype was 0.72 ± 0.15, whereas it was 0.63 ± 0.11 for the C/T genotype (Table 5).

Posttreatment serum TB: Atorvastatin significantly elevated the TB level (mg/dL) to approximately equal values posttreatment in both C/T and T/T carriers (p values for the t test were < 0.001) (Table 6). Serum TB levels were elevated from 0.63 ± 0.11 to 0.86 ± 0.20 and from 0.72 ± 0.15 to 0.87 ± 0.23 in C/T and T/T carriers, respectively. However, in the case of the CYP3A4*1B wild-type genotype (C/C), the TB increase from 0.65 ± 0.07 to 0.81 ± 0.13 was not statistically significant (t test P value > 0.05) (Table 6).

**Table 6:** Variations in serum TB levels (mg/dL) based on genotype before and after atorvastatin treatment for one month

Genotype (n)	Serum TB^a^ level (mg/dL)		Mean difference (95% CI^c^)	P value^†^ (2-tailed)
	Pretreatment (Mean ± SD)^b^	Posttreatment (Mean ± SD)^b^		
CYP3A4*1B (C/C) (n = 2)	(0.65 ± 0.07)	(0.81 ± 0.13)	0.16 (− 0.42, 0.73)	0.18
CYP3A4*1B (C/T) (n = 24)	(0.63 ± 0.11)	(0.86 ± 0.20)	0.23 (0.17, 0.30)	< 0.001*
CYP3A4*1B (T/T) (n = 74)	(0.72 ± 0.15)	(0.87 ± 0.23)	0.15 (0.12, 0.20)	< 0.001*
CYP3A5*3 (T/C) (n = 32)	(0.63 ± 0.12)	(0.82 ± 0.28)	0.19 (0.11, 0.28)	< 0.001*
CYP3A5*3 (C/C) (n = 67)	(0.72 ± 0.14)	(0.89 ± 0.19)	0.17 (0.13, 0.20)	< 0.001*

^a^TB: total bilirubin

^b^Values are given as the mean (standard deviation (SD))

^c^95% CI: 95% confidence interval

^†^Paired t test

^*^P value is considered significant

Serum TB percentage increase: The posttreatment TB percentage increase was greater in the C/T genotype group (38.26 ± 22.79) than in the T/T genotype group (22.44 ± 23.17, P value < 0.05) (Table 5). Pearson’s correlation revealed a weak positive correlation between the posttreatment TB percentage increase and genotype (C/T) ((r = 0.30), (n = 24), and (P value < 0.05)). However, the correlation between the posttreatment TB percentage increase and the genotype (T/T) was weak and indirect (r = − 0.30, (n = 74) (P value < 0.05)) (Table 3).

##### Serum CK

Baseline serum CK: The (C/C) genotype carriers of the CYP3A4*1B SNP had higher baseline CK values (U/L) than did the patients in the groups carrying the (C/T) and (T/T) genotypes (P value < 0.001). The baseline CK value concerning the CYP3A4*1B (C/C) genotype was 159 ± 110.31. On the other hand, the baseline CK values for the C/T and T/T genotypes were 64.96 ± 28.36 and 74.35 ± 27.03, respectively (Table 5).

Changes in the percentage of serum CK: The posttreatment CK percentage increase was greater in both the CYP3A4*1B (C/T) and (T/T) genotype carriers than in the C/C genotype carriers (P value < 0.05). The levels were 12.32 ± 18.68 and 18.77 ± 19.39 for the (C/T) and (T/T) genotype carriers, respectively, and 25.50 ± 57.02 for the (C/C) genotype carriers (Table 5).

**Fig. 2:**
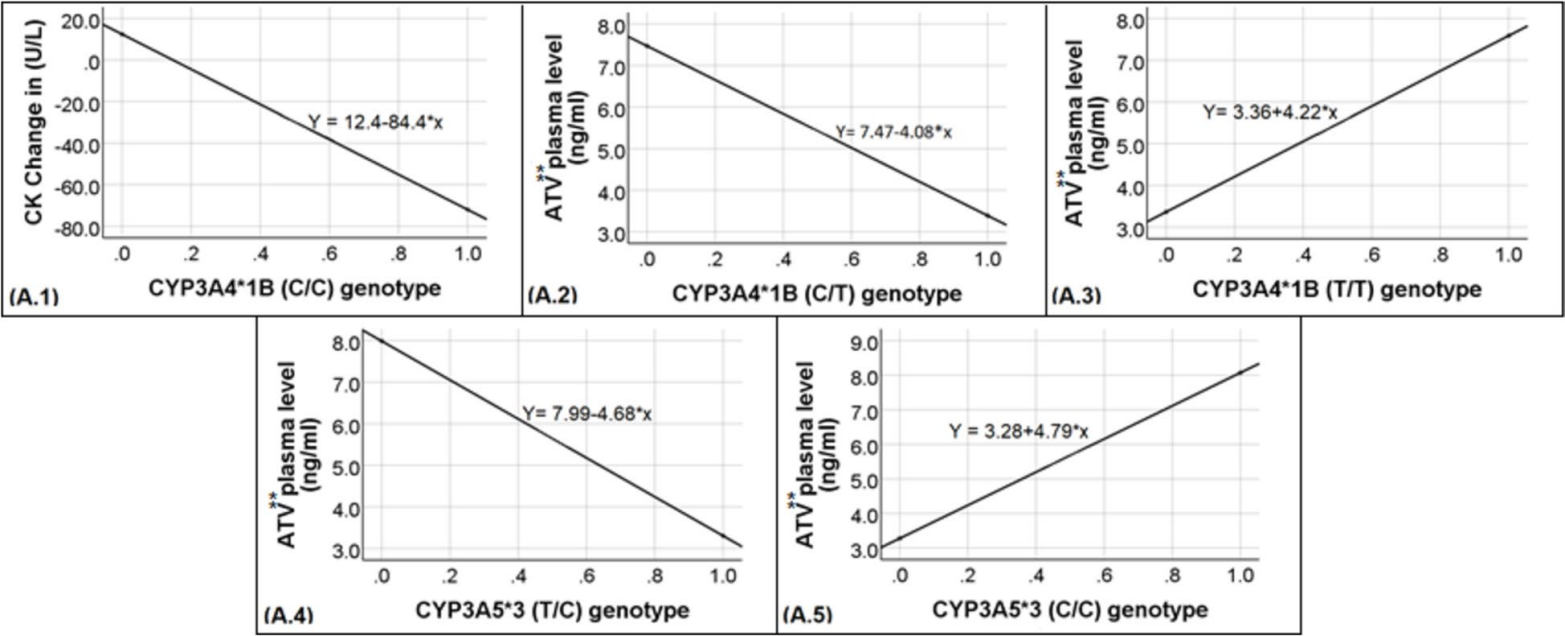
Graphical representations of the statistical regression analysis results. Plots (A.1) to (A.5) show simple linear regression analysis and indicate the direction of the relationship between (X) (predictor) variables and (Y) (dependent) variables as unstandardized predicted values. Plot (A.1) reveals the relationship between the CYP3A4*1B (C/C) genotype and the change in the posttreatment CK (U/L). Plot (A.2) demonstrating the relationship between the CYP3A4*1B (C/T) genotype and the plasma atorvastatin concentration (ng/ml). Plot (A.3) showing the relationship between the CYP3A4*1B (T/T) genotype and the plasma atorvastatin concentration (ng/ml). Plot (A.4) showing the relationship between the CYP3A5*3 (T/C) genotype and the plasma atorvastatin concentration (ng/ml). Plot (A.5) illustrates the relationship between the CYP3A5*3 (C/C) genotype and the plasma atorvastatin concentration (ng/ml). **ATV: Atorvastatin

Pearson’s correlation revealed significant correlations between the change in CK levels posttreatment and both genotypes of CYP3A4*1B (C/C) and (T/T) (P values < 0.001 and < 0.05, respectively). With respect to the (C/C) genotype, there was a moderate, negative correlation (r = − 0.60) (n = 2). For the (T/T) genotype, there was a weak, direct correlation (r = 0.30, (n = 74)) (Table 3). Furthermore, linear regression analysis revealed a significant relationship between CYP3A4*1B (C/C) (as a predictor) and the change (decrease in this genotype) in the posttreatment CK (as the outcome). The regression coefficient β value was − 84.40 (P < 0.001) (Table 4 and Fig. 2).

#### The CYP3A5*3 (*rs776746* T/C) genetic variant

The relationships between the CYP3A5*3 (*rs776746* T/C) variant and ALT, AST, total bilirubin (TB), and creatine kinase (CK) levels were explored.

##### Baseline liver enzymes/serum TB

The baseline liver enzymes and TB levels were greater in the genotype (C/C) carriers than in the genotype (T/C) carriers (P value < 0.05). The baseline ALT level in the C/C genotype carriers was greater (19.48 ± 4.96) than that in the T/C genotype individuals (17.09 ± 4.00) (P value < 0.05). The baseline AST levels were greater in the C/C genotype group (21.51 ± 7.93) than in the T/C genotype group (17.66 ± 4.19) (P value < 0.05). The baseline TB levels were greater in the C/C genotype group (0.72 ± 0.14) than in the T/C genotype group (0.63 ± 0.12) (P value < 0.05) (Table 5).

##### Posttreatment serum TB

Atorvastatin significantly increased the levels of TB (mg/dL) after the four-week treatment in both the T/C and C/C groups (Table 6). The levels increased significantly from 0.63 ± 0.12 to 0.82 ± 0.28 and from 0.72 ± 0.14 to 0.89 ± 0.19 in the T/C and C/C carriers, respectively. The P values for the t test were < 0.001 (Table 6).

##### Serum CK

There was no evidence of a difference between the genotypes (C/C and T/C) regarding the baseline or posttreatment serum levels of CK (P value > 0.05) (Table 5).

### Atorvastatin plasma level

#### Chromatography and selectivity

**Fig. 3:**
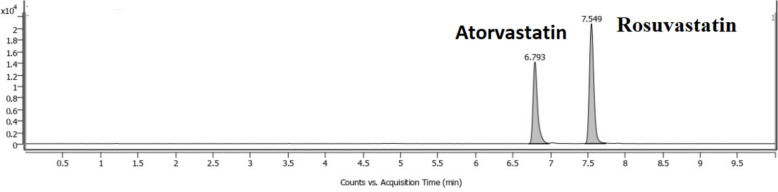
MRM transitions of atorvastatin and rosuvastatin (IS). After 18 h of oral administration of 40 mg atorvastatin, the sample was collected from a participant in this study

Figure 3 displays the MRM transitions of atorvastatin and the IS in an Egyptian patient's LC‒MS/MS chromatogram following an 18-h oral dose of 40 mg atorvastatin. The atorvastatin and IS retention durations were approximately 6.8 and 7.5 min, respectively. The IS and atorvastatin peaks were clearly differentiated. Within the 10-min run period, no endogenous chemical or medication was found to significantly interfere with the atorvastatin and IS retention times. The observed IS and atorvastatin retention times did not significantly change throughout the three-month validation period (RSD < 1.0%).

#### LC‒MS/MS validation

*Linearity:* The AUP ratio of atorvastatin to the IS in the plasma showed excellent linear associations (r = 0.998) for the 1–1000 ng/mL concentration range. With a mean correlation of 0.998 ± 0.001, the AUP ratios (y) against atorvastatin concentrations (x) had a mean linear regression equation of Y =  − 0.03517 + 0.01784X.

*Sensitivity and carry-over:* The lower limit of quantification (LLOQ) of this assay for human plasma was one ng/mL, and the corresponding RSD of the ultrafiltrate was 9.6%. At a signal‒to-noise ratio (S/N) > 3, the LOD was 0.3 ng/mL. No indication of sample carryover from one run to the next was found.

*Accuracy and precision:* By comparing the linear regressions of three standard plots created on three separate days over three months, the reproducibility of the assay was assessed. For each of the three slopes, the RSD was 11.7%, and the mean correlation coefficient was > 0.996. The intra- and interday measurements, as well as the slopes of the calibration curves, did not differ significantly (p > 0.05) according to the ANOVA results. The results validated the reproducibility of the assay method. A value of 10% was established as the highest allowable limit for accuracy and precision. The precision (RSD) within and between runs was less than 10%. For atorvastatin, the accuracy as a relative error was 4.8 ± 1.7.

*Extraction recovery:* Atorvastatin recovery was 89.8 ± 8.0% on average, with an RSD ≤ 10.5%. Over the range of concentrations examined, there was no discernible variation in the extraction effectiveness of the current assay.

*Matrix effect:* By comparing spiked samples (after processing) with spiked injection solvents, the matrix effect was evaluated; the results revealed a difference of less than 10%.

*Stability:* Processed samples kept for 24 h at 10 °C in the autosampler were stable for both the IS and atorvastatin, with mean estimated values falling between 8.7% and 10% of the nominal concentration.

#### Clinical applications involving patient plasma samples

LC‒MS/MS technology can be used to determine the plasma atorvastatin concentration with satisfactory results. The technique demonstrated high sensitivity in properly quantifying clinical samples, spanned the therapeutic range of observed concentrations, and demonstrated excellent clinical application. Following atorvastatin treatment, the plasma levels of all the study participants (n = 100) were measured via LC‒MS/MS at the same intervals before the next dose. Table 5 shows that the plasma levels of atorvastatin had a mean value of 6.49 ng/ml and a standard deviation of 3.00 ng/ml.

#### CYP3A4*1B genotypes and plasma levels of atorvastatin

Atorvastatin plasma levels (in ng/ml) were greater in carriers of the T/T genotype than in carriers of the other genotypes (C/T) and (C/C) (P value < 0.05). The plasma levels were 7.59 ± 2.69 for the T/T genotype and 3.39 ± 1.03 and 3.08 ± 0.82 for the C/T and C/C genotypes, respectively (Table 5).

Pearson’s correlation analysis revealed significant correlations (P values < 0.001) between the plasma atorvastatin concentration (in ng/ml) and both the CYP3A4*1B (C/T) and (T/T) genotypes. With respect to the (C/T) genotype, there was a moderate, inverse relationship (r = − 0.60, n = 24). For the (T/T) genotype, there was a moderate, direct relationship (r = 0.62, n = 74) (Table 3).

Simple linear regression was used to explore the relationships of C/T and T/T genotypes with the continuous dependent variable atorvastatin plasma levels (in ng/ml). The regression coefficients β were − 4.08 and 4.22 for the C/T and T/T genotypes, respectively (P < 0.001) (Table 4 and Fig. 2).

Logistic regression as a prediction model explained the significant relationship between the CYP3A4*1B (T/T) genotype as the dependent binary variable and the plasma atorvastatin concentration (in ng/ml) as the predictor variable. The OR was 1.96 (95% CI: 1.50–2.6, P < 0.001) (Table 4).

### CYP3A5*3 genotypes and plasma levels of atorvastatin

Patients with the C/C genotype had higher plasma atorvastatin levels (8.07 ± 2.33) than did those with the T/C genotype (3.30 ± 1.00) (P value < 0.001) (Table 5).

Pearson’s analysis revealed statistically significant correlations (P values < 0.001) between atorvastatin plasma levels (in ng/ml) and both genotypes of CYP3A5*3 (T/C) and (C/C). There was a strong indirect relationship between the T/C genotype and disease severity (r = − 0.73, n = 32). For the (C/C) genotype, there was a strong and direct relationship (r = 0.80, n = 67) (Table 3).

Linear regression analysis revealed the relationships of the T/C and C/C genotypes with the outcome variable, the plasma atorvastatin concentration. The regression coefficients β were -4.68 and 4.79 for the T/C and C/C genotypes, respectively (P < 0.001) (Table 4 and Fig. 2).

The predictive statistical analysis (logistic regression) as an extrapolation model elucidated the significant relationship between the outcome binary variable (C/C) genotype and plasma atorvastatin levels as the independent variable. The OR was 2.43 (95% CI: 1.8–3.3, P < 0.001) (Table 4).

#### ROC curves

ROC curves were used to assess the extrapolative performance of the plasma atorvastatin concentration. Curves were used to assess the clinical value of this prognostic model for detecting carriers of the homozygous mutant genotypes (T/T) and (C/C) of the variants CYP3A4*1B and CYP3A5*3, respectively.

**Fig. 4:**
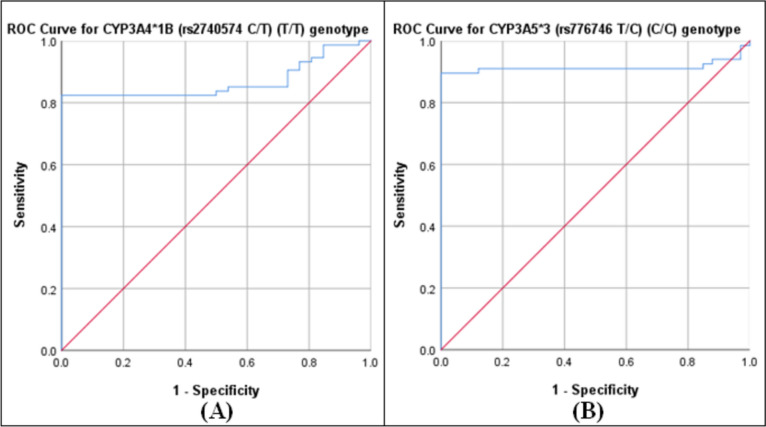
Receiver operating characteristic (ROC) curves. The plots illustrate the predictive performance of the atorvastatin plasma concentration in detecting the CYP3A4*1B (rs2740574 C/T) (T/T) genotype, “ROC curve A”, and the CYP3A5*3 (rs776746 T/C) (C/C) genotype, “ROC curve B.”

The areas under the ROC curves (AUCs) for predicting the CYP3A4*1B (T/T) and CYP3A5*3 (C/C) genotypes were 0.867 (95% CI: 0.797–0.938) and 0.914 (95% CI: 0.849–0.979), respectively. The balanced sensitivity and specificity at the cutoff points yielding the highest Youden index values were calculated (Table 7 and Fig. 4).

**Table 7:** Predictive performance of the plasma atorvastatin concentration in the study subjects (n = 100)

The predicted Genotype	Gene	AUC^a^ (95% CI^b^)	Sensitivity^c^	Specificity^c^	Cut off point
(T/T)	CYP3A4*1B (rs2740574 C/T)	0.867 (0.797- 0.938)	0.824	1	≥ 5.531
(C/C)	CYP3A5*3 (rs776746 T/C)	0.914 (0.849- 0.979)	0.896	1	≥ 6.444

^a^AUC: area under the receiver operating characteristic curve, ^b^CI: confidence interval, ^c^Balanced sensitivity and specificity at the cutoff point, yielding the maximum Youden index (J) value (sensitivity + specificity − 1)

### Clinical pharmacokinetics of atorvastatin

**Fig. 5:**
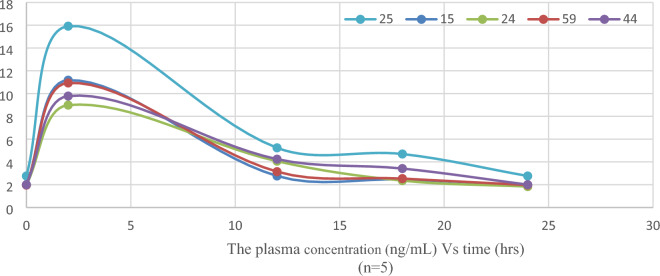
Plasma concentration‒time profile. Atorvastatin pharmacokinetics were determined in five carriers of the genotype (T/T) of the variant CYP3A4*1B (rs2740574 C/T) and the genotype (C/C) of the variant CYP3A5*3 (rs776746 T/C). Samples were collected predose and at two hours, twelve hours, eighteen hours, and twenty-four hours after 40 mg of atorvastatin was administered orally

This method was then successfully used to investigate the clinical pharmacokinetics of atorvastatin. The pharmacokinetics were studied in five carriers of the genotype (T/T) of the variant CYP3A4*1B (*rs2740574* C/T) and the genotype (C/C) of the variant CYP3A5*3 (*rs776746* T/C). The plasma concentration‒time profile of 40 mg atorvastatin after oral administration is shown in Fig. 5. The main pharmacokinetic parameters of atorvastatin are shown in Table 8.

**Table 8:** Comparison between the pharmacokinetic parameters of carriers of the genotype (T/T) of the variant CYP3A4*1B (rs2740574 C/T) and the genotype (C/C) of the variant CYP3A5*3 (rs776746 T/C) and the pharmacokinetic parameters reported in other studies

Human Sample (n) Parameter	The Carriers of the investigated genotypes (Arab Egyptians) (n = 5)			Arab population		Caucasian population						Asian population			
	Mean (SD)^a^	Median (IQR)^a^	GM^a^ (CV%)^a^	Egyptian Volunteers (n = 12) [45]	Jordanians (n = 18) [46]	Americans (n = 25) [47]	Americans (n = 30) [25]	Americans (n = 16) [48]	Finns (n = 12) [49]	Germans (n = 24) [50]	Swedes (n = 42) [51]	Chinese (n = 31) [25]	Japanese (n = 30) [25]	Koreans (n = 60) [52]	Pakistanis (n = 18) [53]
AUC^b^, (ng.h/mL)	126.34 (20.47)	126.34 (106.53–146.15)	125.08 (16%)	122.74 (7.48) GM^a^ (SD)^a^	120.00 (44.12) Mean^a^ (SD)^a^	80.80 (49.20) Mean (CV%)^a^	72.70 (61.00–86.80) GM^a^ (95% CI)^a^	101.30(57.20) Mean (CV%)^a^	61.40 (36.20) Mean (SD)^a^	103.25 (63.44) Mean (SD)^a^	61.60 (22.10 − 158.40) GM^a^ (min − max)^a^	111.00 (93.70–133) GM^a^ (95% CI)^a^	123.00 (103–147) GM^a^ (95% CI)^a^	150.32 (69.85) Mean^a^ (SD)^a^	73.95 (1.72) Mean)SEM)^a^
(p value)^g^	NA^h^			0.83	0.58	0.02*	0.01*	0.09	0.01*	0.11	< 0.05*	0.24	0.85	0.10	0.01*
t½^c^, (h)	13.98 (3.58)	13.03 (10.78–17.65)	13.62 (23%)	5.06 (0.79) GM^a^ (SD)^a^	NR^i^	6.90 (4.10–9.00) Median (IQR)^a^	7.08 (45.90) GM^a^ (CV%)^a^	9.80 (27.10) Mean (CV%)^a^	7.80 (3.60) Mean (SD)^a^	10.18 (7.05–17.40) Median (min–max)^a^	NR^i^	7.85 (39.30) GM^a^ (CV%)^a^	7.47 (51.40) GM^a^ (CV%)^a^	6.04 (1.53) Mean^a^ (SD)^a^	6.65 (0.16) Mean)SEM)^a^
(p value)^g^	NA^h^			< 0.05*	NA^h^	0.01*	< 0.05*	0.06	0.02*	0.04*	NA^h^	0.01*	0.01*	0.01*	0.01*
K^d^, (h^−1^)	0.05 (0.01)	0.05 (0 .04–0.06)	0.05 (24%)	0.14 (0.02) GM^a^ (SD)^a^	NR^i^	0.10‬†	0.10†	0.07†	0.09‬†	0.07†	NR^i^	0.09†	0.09†	0.12†	0.11 (0.003) Mean)SEM)^a^
(p value)^g^	NA^h^			< 0.05*	NA^h^	< 0.05*	< 0.05*	0.03*	< 0.05*	0.09	NA^h^	0.01*	0.01*	< 0.001*	< 0.05*
CL/F^e^, (L/h)	327.06 (58.08)	351.18 (278.24–363.82)	322.208 (16%)	325.89 (20.02) GM^a^ (SD)^a^	333.33†	495.05†	550.21†	539.60 (64.9) Mean (CV%)^a^	651.47†	447.70 (120–1151) Median (min–max)^a^	649.35†	360.36†	325.20†	266.10‬†	540.87†
(p value)^g^	NA^h^			0.91	0.82	< 0.05*	< 0.05*	< 0.05*	< 0.001*	< 0.05*	< 0.05*	0.28	0.92	0.08	< 0.05*
CL^f^, (L/h/kg)	0.41(0.07)	0.44 (0.35–0.46)	0.41 (18%)	0.53†	0.51†	0.87†	0.90†	0.80†	1.16†	0.79‬†	1.03‬†	0.65†	0.57†	0.50†	NR^i^
(p value)^g^	NA^h^			0.04*	0.04*	< 0.001*	< 0.05*	< 0.001*	< 0.001*	< 0.001*	< 0.05*	0.01*	0.02*	0.054	NA^h^

^a^Pharmacokinetic data are expressed in other studies as the mean (standard deviation (SD)), median (interquartile range (IQR)), geometric mean (GM) (coefficient of variation (CV%)), GM (SD), mean (CV%), GM (95% confidence interval (95% CI)), median (minimum value − maximum value (min–max)), GM (min − max), or mean (standard error of the mean (SEM)), ^b^(AUC): Area under the curve, ^c^(t½): the terminal plasma half-life, ^d^(k): elimination rate constant, ^e^(CL/F): apparent oral clearance, ^f^(CL): Drug clearance, ^g^A one-sample t-test was conducted to compare reported means. A one-sample median test was used to compare reported medians with the pharmacokinetic data of this study, ^h^(NA): not applicable, ^i^(NR): not reported

^†^ Calculated value from the reported parameters, k = 0.693/t½, CL/F = Dose/AUC, CL = Dose. F/AUC, oral bioavailability (F) = 0.12[89–91], CL corrected for body weight (L/h/kg) = CL/mean reported weight in kilograms

^*^Statistically significant

## Discussion

The findings linking *CYP3A4* and *CYP3A5* to the response to atorvastatin are not entirely consistent [3, 7–12, 14–18]. Some studies have associated polymorphisms of these enzymes with positive clinical consequences after atorvastatin therapy, and other studies have associated them with negative therapeutic outcomes [3, 7–12, 14–18]. These dissimilar responses are significantly apparent among different populations [45–47]. The observed variation in results may be attributed to the influence of ethnic diversity on the response to medications [48]. It is imperative to consider ethnic diversity when interpreting and implementing pharmacogenomic findings in clinical practice [48]. In this context, the impacts of genetic variations in *CYP3A4* (*rs2740574* C/T) and CYP3A5*3 (*rs776746* T/C) on the response to atorvastatin treatment have not been previously studied among Egyptians [7]. Accordingly, this study investigated the effects of these genetic polymorphisms on atorvastatin therapy among the Egyptian population. This research revealed significant associations between genetic variations in *CYP3A4* (*rs2740574* C/T)/CYP3A5*3 (*rs776746* T/C) and the response to atorvastatin therapy.

### Allele frequencies of the SNPs among the study Egyptian participants

Both alleles C and T of the variant *rs2740574* in the *CYP3A4* gene have yet to be examined in the Egyptian population (Arab population). This study revealed a high frequency of the CYP3A4*1B variant allele (T) in Egyptian participants, similar to previous findings in the Jordanian population [49].

Similarly, the findings indicate a high frequency of the CYP3A5*3 variant allele (C) among Egyptian study subjects, which is consistent with previous reports from research involving Egyptian volunteers [50]. Additionally, the homozygous mutant genotype of the CYP3A5*3 variant is prevalent, and the frequency of this variant is predominant among 76 Egyptian kidney transplant patients [51].

Therefore, the allelic frequencies of the CYP3A4*1B and CYP3A5*3 variant alleles were widespread among the Egyptian study subjects.

### Effect of genetic polymorphisms on atorvastatin effectiveness

#### The CYP3A4*1B (*rs2740574* C/T) genetic variant

The results of this study revealed that the change in the serum TG concentration after atorvastatin therapy was affected by the CYP3A4*1B variant. The decrease in the serum TG percentage was more remarkable in the carriers of the variant allele (T) of CYP3A4*1B ((C/T) and (T/T) individuals) than in the carriers of the homozygous wild-type genotype (C/C). Hence, this relationship is considered to be close and comparable to that reported in research on 142 hypercholesterolemic Chilean patients [3]. This Chilean study attributed the substantial improvement in lipid and lipoprotein profiles after four weeks of atorvastatin treatment to the CYP3A4*1B (*rs2740574*) SNP, which reduces the activity of *CYP3A4* and enhances the efficacy of atorvastatin [3].

#### The CYP3A5*3 (*rs776746* T/C) genetic variant

Serum TG reduction after atorvastatin therapy was affected by the CYP3A5*3 (*rs776746* T/C) variant. This significant reduction was greater in the homozygous mutant genotype (C/C) carriers than in the C/T genotype carriers. Our findings were congruent with those of a study in Greek patients, revealing an apparent improvement in the lipid panel in carriers of the variant allele CYP3A5*3 [52]. Furthermore, a study in European Caucasians concluded that the CYP3A5*3 SNP enhanced the response to atorvastatin therapy (P value < 0.05) [9, 10, 14]. Conversely, in a different population, research demonstrated that the CYP3A5*3 (*rs776746*) SNP did not influence the response to atorvastatin in Chilean subjects [3].

From this perspective, the CYP3A5*3 (*rs776746* T/C) variant increased the response to atorvastatin in Egyptians.

### Effect of genetic polymorphisms on the safety of atorvastatin

#### The CYP3A4*1B (*rs2740574* C/T) genetic variant

##### CYP3A4*1B and serum TB

Carriers of the homozygous mutant (T/T) genotype of the variant CYP3A4*1B (*rs2740574* C/T) had greater baseline TB than (C/T) genotype carriers did. Elevated serum TB levels are associated with reduced *CYP3A* enzymatic activity (P < 0.05) [30]. Thus, the greater elevation in baseline TB levels in (T/T) carriers than in (C/T) carriers revealed a more significant reduction in enzymatic activity in the case of the (T/T) genotype than in the (C/T) genotype.

Atorvastatin significantly elevated TB levels after the four-week treatment in both C/T and T/T carriers. The findings of this study are consistent with research reporting an association between atorvastatin therapy and increased TB levels (p < 0.001) [53]. In this context, after the atorvastatin-induced increase in serum TB, there was no evidence of a difference between the increases in serum TB levels in both C/T and T/T genotype subjects. Furthermore, the statistical arithmetic mean values of posttreatment TB levels in both the C/T and T/T genotypes were approximately equal. However, the baseline serum TB concentration was greater in the C/T genotype group than in the T/T genotype group. Consequently, the posttreatment increase in the serum TB percentage was greater in the (C/T) genotype carriers than in the (T/T) carriers.

In light of this, atorvastatin therapy significantly elevated serum TB levels in carriers of the variant T allele of CYP3A4*1B (*rs2740574*).

##### CYP3A4*1B and serum CK

(C/C) Genotype and serum CK: With respect to the (C/C) genotype of the CYP3A4*1B (*rs2740574* C/T) variant, the pretreatment serum CK level was greater than that of the other genotypes. This elevation was attributed to one of the two (C/C) genotype carriers, a 38-year-old male with a body mass index (BMI) of 31 kg/m^2^ (class I obesity). This subject participated in a long-distance running race for weight loss before being recruited for this study. This (C/C) carrier has not returned to this strenuous physical exercise since he entered the research. Accordingly, this patient’s pretreatment serum CK level was elevated (237 U/L) because of physical activity. This high level elevated the mean value of the baseline serum CK in the (C/C) genotype carriers more than in the other genotypes.

Furthermore, both the postatorvastatin treatment change and percentage change in the serum CK levels in both the C/T and T/T genotypes were greater than those in the C/C genotype. This significant difference was due to the cessation of strenuous exercise in the case of the (C/C) genotype carrier. This interpretation is consistent with a study that revealed a significant increase in serum CK levels after long-distance running (P value < 0.001) [54].

CYP3A4*1B genotypes/atorvastatin dose and serum CK: The CYP3A4*1B (*rs2740574* C/T) variant is linked to decreased enzymatic activity, which increases the chance of elevating the plasma level of atorvastatin [3]. Similarly, in the literature, a high plasma concentration of atorvastatin was linked to increased serum CK [55]. However, in the present study, the atorvastatin concentration did not appear to be associated with the seemingly high changes in serum CK in the case of the C/T and T/T genotypes. Regarding the (T/T) genotype, the atorvastatin concentration was more than twice as high as the concentration in the case of the (C/T) genotype. Conversely, there was no evidence of a difference between these two genotypes in either the change in or the percentage change in the serum CK concentration. These findings are consistent with a study that reported no association between CYP3A4*1B genotypes and high serum CK levels (P value > 0.05) [17]. In addition, in this study, the participants adhered to atorvastatin 40 mg. Nonetheless, atorvastatin at this dose was not high enough to be linked to the apparent serum CK level in the case of the C/T and T/T genotypes. This finding was consistent with research reporting a significant increase in posttreatment serum CK levels with increasing atorvastatin dose (80 mg) (P value < 0.05) [55].

Therefore, there was no evidence of a relationship between CK elevation and the CYP3A4*1B genotype or atorvastatin at a dose of 40 mg.

#### The CYP3A5*3 (*rs776746* T/C) genetic variant

In this study, the homozygous mutant genotype (C/C) carriers of the CYP3A5*3 (*rs776746* T/C) variant presented higher baseline liver enzymes and TB levels than did the T/C genotype carriers. Previous research has shown that carriers of the (C/C) genotype are *CYP3A5* nonexpressors [14, 56]. In this context, a study reported that high ALT and TB levels were associated with decreased *CYP3A* enzymatic activity (P < 0.05) [30]. The increased elevation in the baseline liver enzymes and TB values in the C/C carriers subsequently resulted in a more significant reduction in enzymatic activity.

Serum TB levels were significantly increased after four weeks of atorvastatin treatment in both the T/C and C/C groups. Our results were concordant with research that revealed a link between atorvastatin therapy and elevated TB levels (p < 0.001) [53]. However, after the significant atorvastatin-induced increase in serum TB, there was no evidence of a difference between the increases in serum TB levels in both T/C and C/C genotype carriers.

Accordingly, atorvastatin therapy significantly elevated serum TB levels regardless of the genotype of CYP3A5*3 (*rs776746* T/C).

### Atorvastatin plasma level

Notably, atorvastatin levels (ng/ml) were greater in homozygous mutant genotype (T/T) carriers than in carriers of other genotypes. This increase could be attributed to the reduction in *CYP3A4* metabolic activity caused by the CYP3A4*1B variant [3]. In light of this, the genotype (T/T) of the CYP3A4*1B (*rs2740574* C/T) variant was associated with high atorvastatin plasma levels (ng/ml).

The carriers of the C/C genotype had prominently greater atorvastatin levels (ng/ml) than did those of the T/C genotype. This finding confirmed reports from the literature that subjects with the (C/C) genotype are nonexpressers of the metabolic enzyme *CYP3A5* [14, 56]. Accordingly, the genotype (C/C) of the CYP3A5*3 (*rs776746* T/C) SNP was linked to elevated plasma atorvastatin levels (ng/ml).

#### ROC curves

This study revealed that plasma atorvastatin levels had a substantial predictive effect on homozygous mutant genotypes (T/T) and (C/C) in carriers of the SNPs CYP3A4*1B (*rs2740574* C/T) and CYP3A5*3 (*rs776746* T/C). The probabilities of atorvastatin plasma levels correctly detecting the (T/T) and (C/C) genotypes of CYP3A4*1B and CYP3A5*3 were good and excellent, respectively. This apparent predictive performance may be attributed to the significant relationships between these genotypes (dependent binary variables) and plasma atorvastatin concentrations (predictor variables), as elucidated in this study by another predictive statistical analysis (logistic regression). Predicting these genotypes could be helpful in the case of barriers and challenges for the clinical application of genotyping. To our knowledge, no research has reported such a predictive statistical analysis.

Within this context, predicting the genetic polymorphisms of CYP3A4*1B and CYP3A5*3 has crucial clinical consequences. For instance, these polymorphisms influence the pharmacokinetics of statins [7, 10, 57]. Furthermore, both the CYP3A4*1B and CYP3A5*3 genotypes are vital for adjusting the dose of the immunosuppressant tacrolimus in the maintenance therapy stage after kidney transplantation [58, 59]. In addition, regarding CML, the response to the targeted cancer drug imatinib is associated with the CYP3A5*3 genotypes [60].

From this perspective, predicting CYP3A4*1B and CYP3A5*3 homozygous mutant genotypes with high sensitivity and specificity could help confront challenges in implementing genotyping. In addition, this prediction could have critical clinical significance if personalized medicine is applied.

### Atorvastatin pharmacokinetics

Atorvastatin clinical pharmacokinetics were evaluated in Egyptians who carried both genotypes (T/T) and (C/C) of the SNPs CYP3A4*1B (*rs2740574* C/T) and CYP3A5*3 (*rs776746* T/C), respectively. Both genetic variants decreased the metabolic activities of both *CYP3A4* [3] and *CYP3A5* [14, 56]. Therefore, the pharmacokinetics of atorvastatin were significantly affected. The findings of this study were compared with the pharmacokinetics of atorvastatin in various populations (Table 8).

#### Comparison with the Arab population (Egyptians and Jordanians)

Atorvastatin pharmacokinetics were investigated in healthy Egyptian volunteers (without both genetic polymorphisms) [61]. This research revealed that the elimination half-life (t½) of atorvastatin significantly increased to more than double that of healthy Egyptians (P < 0.001). Moreover, the clearance (CL) in this study was significantly lower than that in Egyptian volunteers (P < 0.05) [61]. Furthermore, compared with healthy Arabian‒Asian Jordanian subjects, patients with CYP3A4*1B and CYP3A5*3 genetic polymorphisms had significantly reduced atorvastatin CL (P < 0.05) [62].

#### Comparison to the Caucasian population

##### American subjects

This study showed a significantly greater AUC and t½ (approximately doubled) than did the studies of Kacey Anderson et al. (P < 0.05) and B. K. Birmingham (P < 0.05), which involved American Caucasian subjects [35, 63]. In addition, this study demonstrated significantly less apparent oral clearance (CL/F) (P < 0.05) than the other two studies did [35, 63]. The atorvastatin CL was approximately less than half of what was reported in Kacey Anderson et al.’s research (P < 0.001) and B. K. Birmingham’s research (P < 0.05) [35, 63]. Moreover, the atorvastatin CL/F and CL were significantly lower than those reported in the study by N. Rao et al., which recruited American subjects (P values < 0.05 and < 0.001, respectively) [64].

##### Finnish subjects

Compared with healthy Finnish Caucasians, the genetic variations in CYP3A4*1B and CYP3A5*3 increased both the AUC (almost doubled) (P < 0.05) and t½ (P < 0.05) [65]. Furthermore, this study revealed a lower CL/F (decrease to half) (P < 0.001) and CL (less than half) (P < 0.001) than those reported in studies of Finnish subjects [65].

##### German subjects

Compared with research on German Caucasians, this study revealed greater t½ value (P < 0.05) [66]. In addition, the CL/F was lower than that in German subjects (P values < 0.05) [66]. The CL of atorvastatin was also reduced by half (P values < 0.001) [66].

##### Swedish subjects

In contrast with Swedish Caucasian subjects, this study revealed a greater (nearly doubled) AUC (P < 0.05) [67]. In addition, both CL/F and CL were significantly reduced. Atorvastatin CL/F decreased to half (P < 0.05), and CL decreased to less than half (P < 0.05) of what was observed in Swedes [67].

#### Comparison with the Asian population

##### Chinese and Japanese subjects

The genetic polymorphisms CYP3A4*1B and CYP3A5*3 elevated t½ (P < 0.05) in the participants in this study, which was greater than that in healthy Chinese and Japanese subjects [35]. Moreover, this study revealed fewer CLs (P < 0.05) than those reported for Chinese and Japanese volunteers [35].

##### Korean subjects

The findings revealed higher t½ values (more than doubled) than those reported in research involving healthy Korean Asian subjects (P < 0.05) [68]. On the other hand, there was no evidence of a difference regarding the CL corrected for the arithmetic mean of the reported body weights (P = 0.054) [68]. However, regarding the CL corrected for weights ≤ 63.2 kg in the Korean subjects, the patients in this study had significantly lower CLs (P < 0.05) [68].

##### Pakistani subjects

Compared with M. Sohail et al., the participants in this study had greater AUCs (P < 0.05) and t½ values (more than doubled) (P < 0.05) [69]. Furthermore, the CL/F of patients treated with atorvastatin was significantly lower than that of Pakistani volunteers (P < 0.05) [69].

### Genetic polymorphisms and patients' medical histories

The CYP3A4*1B SNP has been associated with an increased risk of obesity [24–26]. However, this study revealed no evidence of a difference in BMI (kg/m^2^) among the three genotype carriers of the CYP3A4*1B variant. Similarly, the CYP3A5*3 variant has been linked to an increased likelihood of developing hypertension and increased levels of serum TG [28, 29]. Conversely, the study revealed that there was no evidence indicating a difference between individuals carrying the homozygous mutant genotype (C/C) and those carrying the heterozygous genotype (C/T) of the CYP3A5*3 SNP regarding systolic blood pressure, diastolic blood pressure or baseline serum TG levels.

### Potential confounding factors

#### Concomitant medications

While recruiting participants, we ensured that all their concurrent medications would not interact with atorvastatin treatment or affect the analysis or the study objectives. In the same context, none of the study subjects had used lipid-lowering agents for at least one month before this research. This period was defined based on the literature, which suggests restarting a statin after at least two weeks of washout [70]. Additionally, washout periods (at least two weeks) before changing statin treatment regimens are sufficient to render statin-tolerant subjects [71]. In addition, subjects who were treated with insulin therapy were excluded from the study because insulin treatment significantly affects lipid profiles [72].

#### Comorbidities

All the participants were candidates for high-intensity statin treatment and did not suffer any comorbidities that may affect the analysis, such as uncontrolled hypothyroidism, which negatively impacts the lipid profile [73]. All subjects with poor liver function or an uncontrolled clinically significant disease were excluded from the study to control for confounding factors in the analyses.

#### Lifestyle

The consumption of alcohol is associated with a greater likelihood of developing liver disease [74, 75]. Smoking also affects the lipid profile by increasing serum TG levels [76]. Smoking increases the occurrence and frequency of liver disorders [77]. Consequently, to control for confounding factors in the analyses, every participant abstained entirely from alcohol and smoking during the research. Additionally, after vigorous physical exertion, CK levels can rise 30 times above the normal upper limit within one day and slowly decrease over the following week [78, 79]. Consequently, all the participants were instructed to avoid engaging in strenuous physical activity from one week before until the end of the study.

### Limitations

This research was conducted at a single tertiary care, large educational institution, which limits its generalizability to other settings. Medical professionals should consider results from only single-center studies after carefully analyzing and comparing their circumstances with those of the study. The research also required adequate funding. The researchers relied on spending on expenses, which decreased the sample size or the number of participants to be included and screened in this study to only 100 subjects. Increasing the research sample size will ensure greater population representation, leading to more precise and reliable results. In addition, to ensure ethical compliance, recruitment and data collection were confined within the approved time frame set by the ethical committees. Consequently, we had to resort to convenient sampling from the study site to identify eligible patients for the study. However, the majority of the participants in the study were female. Furthermore, the wild-type homozygous (T/T) genotype of the variant CYP3A5*3 (*rs776746* T/C) in Egyptian patients was significantly low. The study’s genotyping information revealed that only one patient carried this wild-type genotype. Consequently, data concerning this genotype were excluded from the data analysis. This exclusion prevented a comprehensive analysis of this genotype.

## Conclusions

This study revealed the predominance of the allelic frequencies of the CYP3A4*1B (rs2740574 C/T) and CYP3A5*3 (rs776746 T/C) variants in Egyptians. Both prevalent SNPs could influence the effectiveness and safety of atorvastatin treatment. High plasma atorvastatin levels were detected in the T/T genotype of the CYP3A4*1B variant and the C/C genotype of the CYP3A5*3 SNP. Similarly, atorvastatin plasma levels had significant predictive performance for determining the genotypes (T/T) and (C/C) of carriers of the SNPs CYP3A4*1B and CYP3A5*3, respectively. Predicting these genotypes could be valuable in the case of challenges for the clinical implementation of genotyping. The clinical pharmacokinetics of atorvastatin were assessed in Egyptians who carried both genotypes (T/T) and (C/C) of the SNPs CYP3A4*1B and CYP3A5*3 (the homozygous mutant genotypes). Both genetic variants significantly affected the pharmacokinetics of atorvastatin compared with those of healthy Egyptians and volunteers of different ethnic groups. To the best of the researchers’ knowledge, the effects of the CYP3A4*1B (*rs2740574* C/T) and CYP3A5*3 (*rs776746* T/C) polymorphisms on atorvastatin efficacy and safety have not been previously investigated among the Egyptian population. The findings from this study can guide physicians toward understanding the effects of both SNPs in response to atorvastatin therapy.

### Recommendations and directions for future research

The funding of large-scale multicenter prospective research will help address the study's limitations. Such research will include almost the exact proportions of male and female subjects and more carriers of the rare, wild homozygous (T/T) genotype of the variant CYP3A5*3 (*rs776746* T/C). Large-scale studies can reveal even subtle effects while ensuring robust statistical power and the representativeness of the results. Similarly, only the 40 mg atorvastatin dose was evaluated. Adequate research funding could enable the study of multiple dose levels to provide insights into dose‒response relationships and optimal dosing on the basis of genotypes.

## Data Availability

Data cannot be shared openly to protect study participant privacy. The datasets generated during and/or analyzed during the current study are available from the corresponding author upon reasonable request.
